# Controlled Release of Epigenetically-Enhanced Extracellular Vesicles from a GelMA/Nanoclay Composite Hydrogel to Promote Bone Repair

**DOI:** 10.3390/ijms23020832

**Published:** 2022-01-13

**Authors:** Kenny Man, Inês A. Barroso, Mathieu Y. Brunet, Ben Peacock, Angelica S. Federici, David A. Hoey, Sophie C. Cox

**Affiliations:** 1 School of Chemical Engineering, University of Birmingham, Birmingham B15 2TT, UK; K.L.Man@bham.ac.uk (K.M.); IXP799@student.bham.ac.uk (I.A.B.); MYB925@student.bham.ac.uk (M.Y.B.); 2 NanoFCM Co., Ltd., Nottingham NG90 6BH, UK; Bpeacock@nanofcm.com; 3 Trinity Centre for Biomedical Engineering, Trinity Biomedical Sciences Institute, Trinity College Dublin, D02 R590 Dublin, Ireland; federica@tcd.ie (A.S.F.); dahoey@tcd.ie (D.A.H.); 4 Department of Mechanical, Manufacturing, and Biomedical Engineering, School of Engineering, Trinity College Dublin, D02 R590 Dublin, Ireland; 5 Advanced Materials and Bioengineering Research Centre, Trinity College Dublin & RCSI, D02 R590 Dublin, Ireland

**Keywords:** extracellular vesicles, bone, hydrogel, drug delivery, tissue engineering, epigenetics

## Abstract

Extracellular vesicles (EVs) have garnered growing attention as promising acellular tools for bone repair. Although EVs’ potential for bone regeneration has been shown, issues associated with their therapeutic potency and short half-life in vivo hinders their clinical utility. Epigenetic reprogramming with the histone deacetylase inhibitor Trichostatin A (TSA) has been reported to promote the osteoinductive potency of osteoblast-derived EVs. Gelatin methacryloyl (GelMA) hydrogels functionalised with the synthetic nanoclay laponite (LAP) have been shown to effectively bind, stabilise, and improve the retention of bioactive factors. This study investigated the potential of utilising a GelMA-LAP hydrogel to improve local retention and control delivery of epigenetically enhanced osteoblast-derived EVs as a novel bone repair strategy. LAP was found to elicit a dose-dependent increase in GelMA compressive modulus and shear-thinning properties. Incorporation of the nanoclay was also found to enhance shape fidelity when 3D printed compared to LAP-free gels. Interestingly, GelMA hydrogels containing LAP displayed increased mineralisation capacity (1.41-fold) (*p* ≤ 0.01) over 14 days. EV release kinetics from these nanocomposite systems were also strongly influenced by LAP concentration with significantly more vesicles being released from GelMA constructs as detected by a CD63 ELISA (*p* ≤ 0.001). EVs derived from TSA-treated osteoblasts (TSA-EVs) enhanced proliferation (1.09-fold), migration (1.83-fold), histone acetylation (1.32-fold) and mineralisation (1.87-fold) of human bone marrow stromal cells (hBMSCs) when released from the GelMA-LAP hydrogel compared to the untreated EV gels (*p* ≤ 0.01). Importantly, the TSA-EV functionalised GelMA-LAP hydrogel significantly promoted encapsulated hBMSCs extracellular matrix collagen production (≥1.3-fold) and mineralisation (≥1.78-fold) in a dose-dependent manner compared to untreated EV constructs (*p* ≤ 0.001). Taken together, these findings demonstrate the potential of combining epigenetically enhanced osteoblast-derived EVs with a nanocomposite photocurable hydrogel to promote the therapeutic efficacy of acellular vesicle approaches for bone regeneration.

## 1. Introduction

The treatment of bone fractures represents a tremendous socioeconomic burden worldwide, with approximately 10 million people in the UK afflicted with musculoskeletal disorders [1]. With a growing and ageing population demand for such treatments is only expected to increase. Autologous bone grafts are considered the current gold standard, however, their use is associated with several issues such as their limited availability and donor site morbidity [2]. The combination of bone graft substitutes with osteoinductive growth factors such as bone morphogenic protein 2 (BMP2) have shown positive clinical results [3,4]. However, the use of supraphysiological BMP2 concentrations can result in severe complications including heterotopic ossification, hematoma, and myelopathy [5,6,7]. Thus, there is a significant demand for novel approaches to regenerate damaged bone, overcoming the limitations of current strategies [8]. Cell-based tissue engineering approaches have shown great promise in recent years, with methods combining osteoinductive biomaterials with mesenchymal stromal cells (MSCs) seen as an attractive bone augmentation strategy [9]. Although encouraging results have been observed, the direct transplantation of MSC-based therapies are associated with numerous complications including their uncontrolled differentiation, immunological rejection, inherent heterogeneity, functional tissue engraftment and neoplasm formation [10,11]. Moreover, the clinical translation of cell-based therapies is hindered by substantial hurdles including relatively high costs, scalability of manufacture, government regulations and ethical issues [12]. Hence, there is a growing interest in utilising cell-free approaches as an alternative to stimulate bone repair.

In recent years, an increased body of evidence has demonstrated the influence of the cells’ secretome on eliciting tropic effects on neighbouring cells in the surrounding microenvironment [13,14]. One of these such factors, extracellular vesicles (EVs), are considered a promising acellular tool for regenerative medicine. EVs are cell-secreted lipid nanoparticles that contain a diverse biological cargo such as nucleic acids, proteins and bioactive molecules [15,16,17], and are integrally involved in intercellular communication. The favourable effects once attributed to cells, are now thought to be partly due to the bioactive factors delivered by EVs [18,19]. Moreover, it has been reported that these naturally-derived nanoparticles are integrally involved in bone development through mediating intercellular communications between osteoblast and osteoclasts [20,21]. Additionally, matrix-bound EVs have been suggested to be essential for endochondral ossification [22,23]. Hence, there have been extensive investigations into harnessing these naturally-derived nanoparticles as an acellular approach to stimulate bone repair, overcoming the numerous translational hurdles associated with cell-based therapies. Several studies have reported the considerable utility of EVs in stimulating osteogenesis [24,25,26]. Although great potential has been shown, there has been intensive research into EV engineering strategies to further enhance the therapeutic efficacy of these nano-sized vesicles for bone augmentation strategies [15].

It has become increasingly apparent that epigenetic regulation plays a critical role in controlling cell fate [27,28]. As such, researchers have investigated harnessing epigenetic modifications to augment the cells differentiation capacity for bone augmentation [29,30,31]. Recent reports have also highlighted the potential to use these epigenetic approaches to enhance EV mineralisation capacity. Specifically, EVs isolated from osteoblasts treated with the histone deacetylase inhibitor (HDACi) Trichostatin A (TSA) were found to elicit significantly enhanced osteoinductive potency, due to enrichment in pro-osteogenic microRNAs and transcriptional regulating proteins [32]. Despite these promising new approaches to enhance EV efficacy, the short half-life of these nanoparticles following systemic administration hinders their therapeutic utility [33]. Additionally, local administration of EVs into the defect has only transient results, often requiring successive injections to be clinically effective [34]. The use of injectable biomaterials to facilitate the delivery of EVs has acquired growing interest to improve their bioavailability in situ [35]. Several studies have reported the controlled release of EVs from biomaterial systems [36,37], however, there are limited investigations regarding the delivery of vesicles from pro-osteogenic materials. Ideally these systems should be deliverable in a minimally invasive manner and enhance vesicle retention in situ to facilitate EV-induced bone formation.

Gelatin methacryloyl (GelMA) is a photosensitive hydrogel widely utilised for several tissue engineering applications due to its biocompatibility, biodegradability and low cost [38,39]. Moreover, the photo-crosslinkable nature of GelMA allows for in situ gelation following injection or 3D printing into complex anatomical structures [40,41]. In recent years, GelMA has been employed as a cell carrier given its hydrated 3D microenvironment as well as its ability to support cellular adhesion and functionality. Due to these advantageous properties, GelMA has been extensively investigated as a biomaterial to support bone formation. Moreover, there has been growing evidence demonstrating the use of GelMA to locally deliver EVs and enhance their bioavailability in vivo [42]. Although their potential as an EV delivery vehicle has been demonstrated, GelMA alone is not osteoinductive, therefore is unable to facilitate EV-induced mineralisation in vivo. Moreover, studies delivering EVs within GelMA rely on the physical entrapment of these nanoparticles within the hydrogel, which is linked to the polymer concentration. Low GelMA concentrations (<5wt%) have been reported to be favourable for bone formation due to its highly permissive environment [43], however, these low wt% hydrogels often lack mechanical strength. Hence, increasing GelMA hydrogel functionality to deliver and support EV-induced bone regeneration is much needed.

The use of nanosilicates has emerged as a promising additive to effectively enhance the physical and biological functionality of biomaterials [44,45]. Laponite (LAP) is an FDA-approved synthetic smectite nanomaterial capable of generating colloidal-like suspensions within an aqueous environment [46]. Dispersions of LAP comprise of disc-shaped nanoparticles 1 nm in thickness and 25 nm in diameter displaying a positive rim charge and negative surface charge, providing a broad-spectrum affinity with bioactive molecules [47,48]. LAP has been increasingly investigated for bone tissue engineering applications due to the osteoinductive properties of its degradation products [48,49]. Furthermore, several studies have reported the influence of LAP in accelerating the gelation of polysaccharide matrices through hydrogen bonds, thus improving hydrogel mechanical properties [49]. This has also been shown to enhance the printability of numerous polymers due to the rheology-modifying capabilities offered by LAP [50,51]. Critically, nanoclay-based hydrogels have been shown to behave as a functional vehicle for drug retention and delivery with preferential clay–protein electrostatic interactions [46], which could be employed to control EV release kinetics in vivo. Therefore, the use of a biocompatible photo-polymerisable GelMA hydrogel combined with the shear-thinning and clay–protein immobilisation offered by LAP, could provide a viable vehicle to control the delivery and release kinetics of pro-osteogenic EVs for bone regeneration.

**Figure 1:**
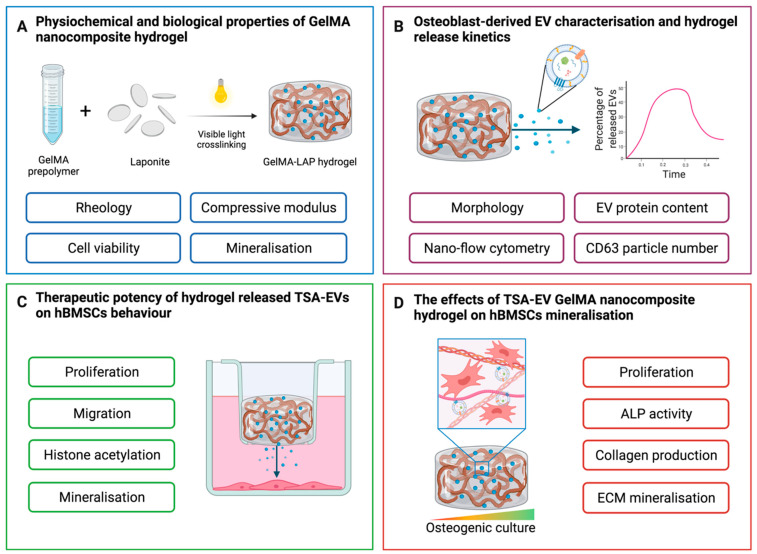
Experimental outline detailing the osteoinductive potency of epigenetically-modified EVs loaded GelMA nanocomposite hydrogel. (**A**) The physiological and biological effects of the GelMA nanocomposite hydrogel was investigated. (**B**) EV isolation, characterisation, and hydrogel-EV release kinetics was assessed. (**C**) The biological efficacy of hydrogel-released EVs on hBMSCs behaviour. (**D**) The influence of TSA-EV functionalised hydrogel on encapsulated hBMSCs mineralisation. Created with BioRender.com (last accessed 13 December 2021).

In this study we investigated the potential of combining epigenetically activated osteoblast-derived EVs (TSA-EVs) with a GelMA nanocomposite hydrogel to promote stem cell mineralisation (Figure 1). The physiochemical and biological influence of LAP incorporation within GelMA was initially evaluated. Osteoblast-derived EVs were characterised and their release kinetics from the nanocomposite hydrogel was assessed via CD63 ELISA. The biological potency of hydrogel-released TSA-EVs on human bone marrow stromal cells (hBMSCs) osteogenic differentiation was evaluated. Finally, the effect of TSA-EVs on encapsulated hBMSCs extracellular matrix mineralisation was investigated.

## 2. Results

### 2.1. Nanosilicate Inclusion Augments GelMA Physicochemical and Osteogenic Properties

**Figure 2:**
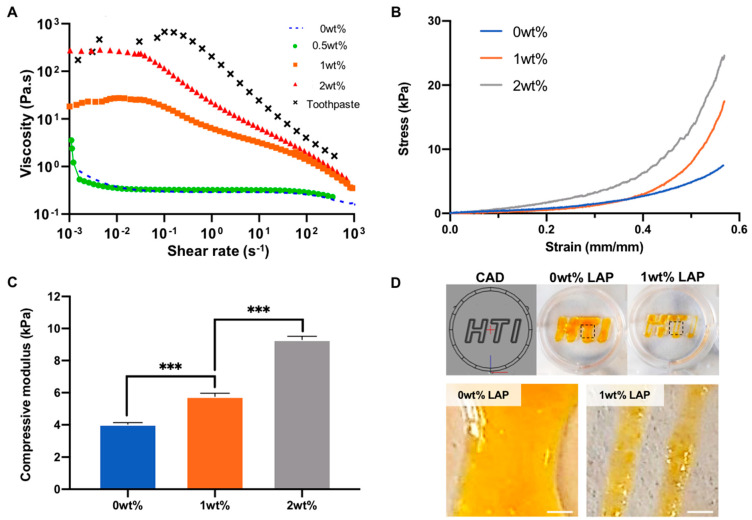
The physicochemical properties of GelMA-LAP hydrogel. (**A**) Viscosity shear rate of pre-polymer solution (GelMA and GelMA loaded with nanosilicates) highlights shear-thinning characteristic of the pre-polymer solution loaded with LAP. (**B**,**C**) Nanocomposite hydrogels were subjected to unconfined compression. From the stress strain curves, the compressive modulus of the hydrogels was calculated from the 0.10–0.20 strain. (**D**) GelMA bioinks were 3D printed to evaluate shape fidelity of printed structures. Scale bar = 1 mm. Data are expressed as mean ± SD (*n* = 3). *** *p* ≤ 0.001.

The influence of LAP incorporation on GelMA physiochemical properties were initially evaluated by assessing the rheological properties. Addition of LAP increased the viscosity of the pre-polymer solution in a concentration-dependent manner (Figure 2A). Moreover, shear-thinning behaviour was observed in groups containing ≥1wt% LAP. Following these initial findings, the compressive modulus of two nanocomposite formulations was assessed. There was a LAP dependent increase in the hydrogel stiffness, with compressive moduli of 4.03 ± 0.09 (0wt%), 5.76 ± 0.2 (1wt%) and 9.3 ± 0.22 kPa (2wt%) (*p* ≤ 0.001) (Figure 2B,C). The influence of LAP on GelMA shape fidelity was evaluated via 3D printing. Our findings showed that LAP-containing bioinks exhibited increased shape fidelity when compared to the LAP-free group (Figure 2D and Supplementary Figure S1).

**Figure 3:**
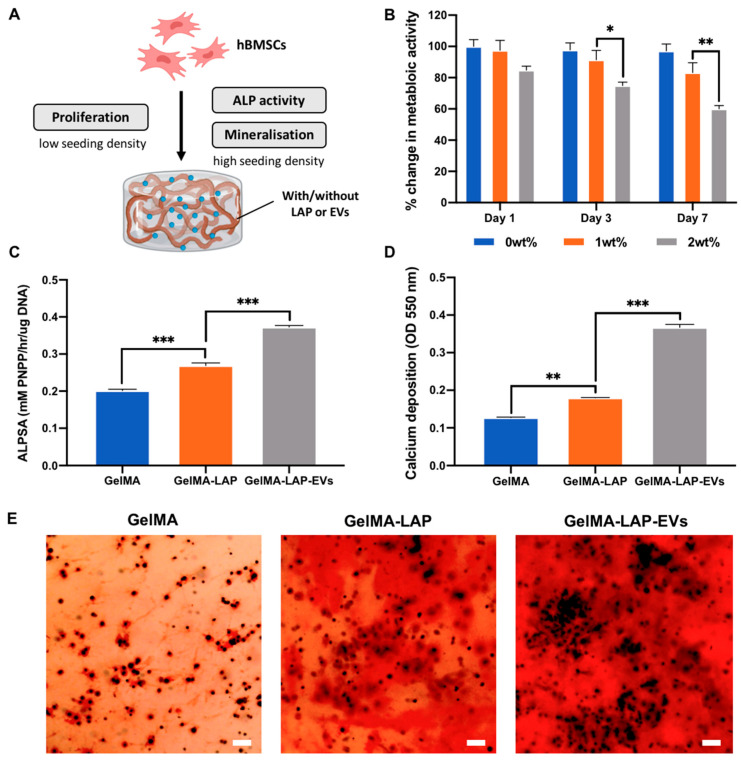
The osteoinductive potency of the GelMA-LAP hydrogel. (**A**) Schematic representation of functional assessments. (**B**) The metabolic activity of hBMSCs within GelMA containing 0, 1 or 2wt% LAP. The effect of LAP and EV loading on hBMSCs (**C**) ALP activity and (**D**,**E**) calcium deposition within the GelMA hydrogel following 2 weeks osteoinductive culture. Black staining indicates mineral nodules. Scale bar = 100 µm. Data are expressed as mean ± SD (*n* = 3). * *p* ≤ 0.05, ** *p* ≤ 0.01 and *** *p* ≤ 0.001.

The influence of LAP on hBMSCs behaviour within the GelMA hydrogel was initially evaluated by assessing proliferation. LAP caused a time-dose dependent reduction on hBMSCs metabolic activity within the hydrogel, with the 2wt% group eliciting a significant reduction in viability compared to the 0 and 1wt% groups on days 3 and 7 of basal culture (Figure 3B) (*p* ≤ 0.05–0.01). Following these initial findings, the osteoinductive potency of 1 wt% LAP within the GelMA hydrogel was evaluated by quantifying alkaline phosphatase (ALP) activity. GelMA-LAP significantly improved encapsulated hBMSCs ALP activity when compared to the LAP-free gel (1.34-fold) (*p* ≤ 0.001) after 2 weeks in osteoinductive culture (Figure 3C). Moreover, EVs derived from untreated osteoblasts (MO-EVs) were incorporated within the composite hydrogel to determine its capacity to promote osteogenesis in this system. EV inclusion further improved hBMSCs ALP activity when compared to the GelMA-LAP (1.38-fold) and the GelMA alone (1.85-fold) group. Similarly, it was observed that LAP incorporation significantly increased hBMSCs calcium deposition when compared to the GelMA alone group (1.41-fold) (*p* ≤ 0.01), with MO-EVs further enhancing the hBMSCs mineralisation capacity when compared to the GelMA-LAP (2.05-fold) and GelMA alone (2.89-fold) groups (Figure 3D,E) (*p* ≤ 0.001).

### 2.2. Characterisation of EVs Derived from TSA Treated Osteoblasts

**Figure 4:**
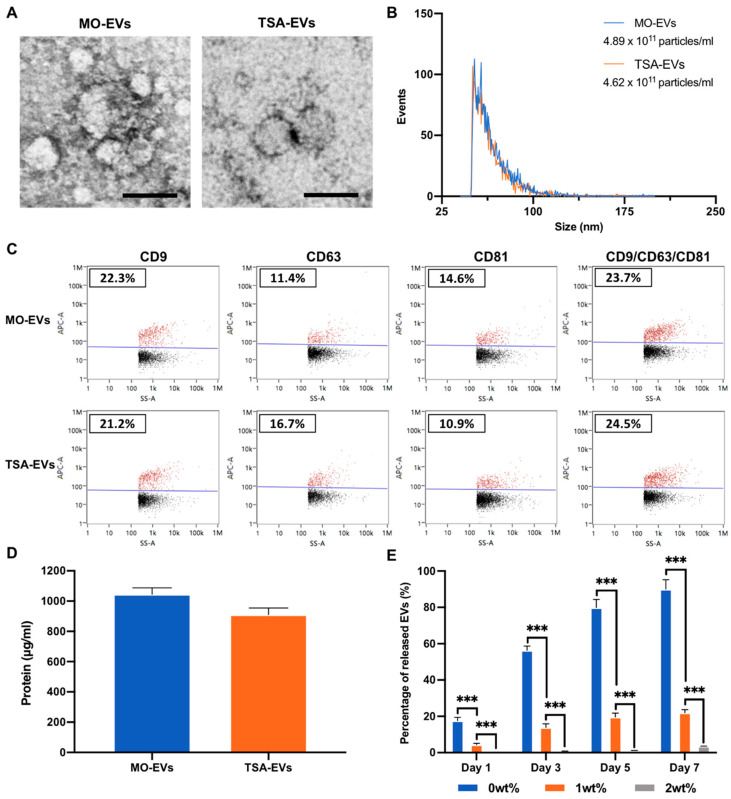
Characterisation of EVs derived from TSA treated and untreated mineralising osteoblasts. (**A**) TEM image of isolated EVs. Scale bar = 50 nm. (**B**) Nano-flow cytometry (NanoFCM) analysis, depicting the size distribution and concentration of particles. (**C**) Single-particle phenotyping of osteoblast-derived EVs. EVs were fluorescently labelled with APC-conjugated antibodies specific to CD9, CD63 and CD81. Bivariate dot-plots of indicated fluorescence versus SSC are shown. In addition, CD9/CD63/CD81 positive particles are shown. (**D**) EV protein content. (**E**) Quantification of EVs released from GelMA hydrogel with/with LAP assessed via CD63 positive ELISA. Data are expressed as mean ± SD (*n* = 3). *** *p* ≤ 0.001.

Differential centrifugation was utilised to isolated EVs from the conditioned media of untreated and TSA-treated osteoblasts over a 2-week culture period. TEM imaging showed both groups displayed particles with a typical size and spherical morphology indicative of nano-sized EVs, where they exhibit heterogeneity in their diameters (Figure 4A). The nano-flow cytometry analysis detected particles with an average diameter of 65.03 ± 14.43 and 62.58 ± 13.06 nm for the MO-EVs and the TSA-EVs respectively (Figure 4B). Single-particle phenotyping analysis was conducted by NanoFCM (Figure 4C). The three tetraspanin markers CD9, CD63 and CD81 were assessed. The MO-EVs particles exhibited a 22.3%, 11.4% and 14.6% positive staining for CD9, CD63 and CD81; while the TSA-EVs elicited 21.2%, 16.7% and 10.9% positive staining for each marker. When assessing triple-positive staining, 23.7% and 24.5% of all particles stained positive for the MO-EVs and the TSA-EVs respectively. The TSA-EVs exhibited a 1.15-fold (*p* > 0.05) reduction in protein content when compared to the MO-EVs (Figure 4D). No significant difference (*p* > 0.05) was observed in the zeta potential of MO-EVs (−7.77 ± 1.53 mV) and TSA-EVs (−6.06 ± 1.06 mV) (Supplementary Figure S2). The EV release kinetics from the nanocomposite GelMA hydrogel was evaluated via CD63 ELISA (Figure 4E). Hydrogels containing LAP released significantly fewer CD63 positive particles when compared to the LAP-free hydrogels over 7 days of culture (*p* ≤ 0.001). At day 1, 17.65 ± 1.78% (0wt%), 4,39 ± 0.74% (1wt%) and 0.10 ± 0.01% (2wt%) of CD63 positive EVs were released. These values increased to 90.12 ± 5.2% (0wt%), 21.96 ± 1.78% (1wt%) and 3.60 ± 0.06% (2wt%) by day 7. Additionally, no significant difference in CD63 positive particle concentration was observed from TSA-EV or MO-EVs loaded GelMA-LAP hydrogels following 7 days incubation (Supplementary Figure S3) (*p* > 0.05).

### 2.3. Hydrogel-Released TSA-EVs Enhance hBMSCs Osteogenic Differentiation

**Figure 5:**
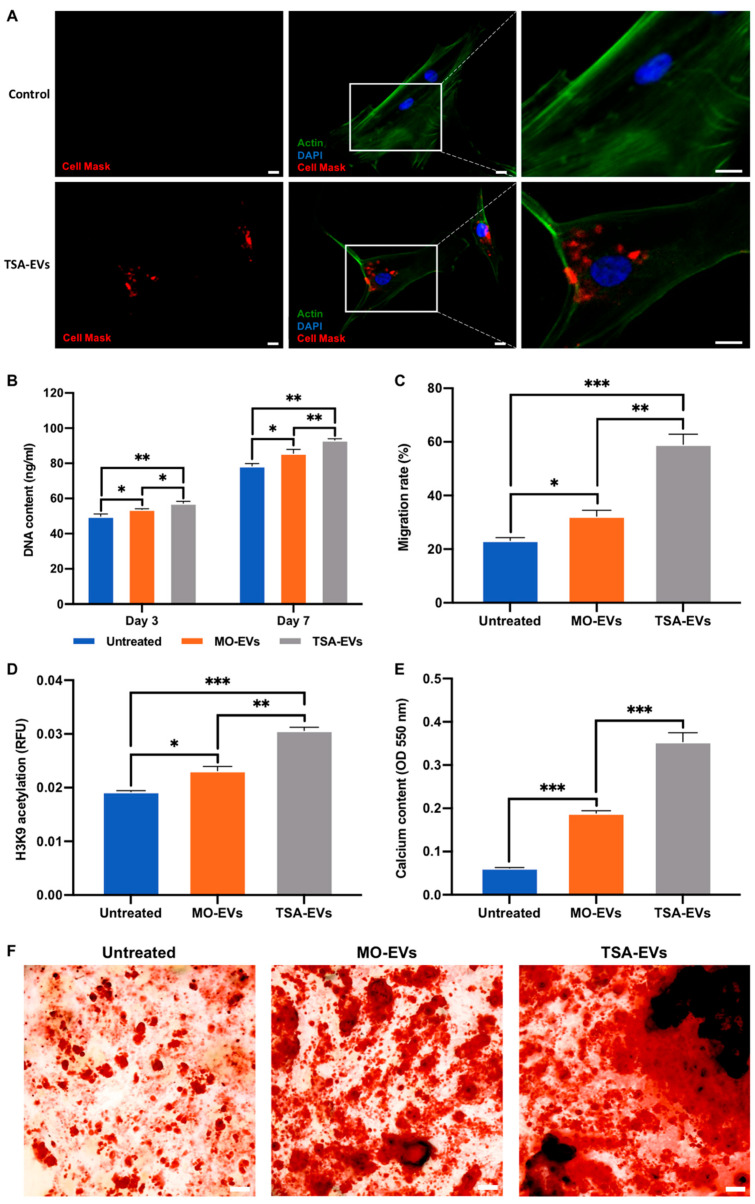
The biological efficacy of hydrogel-released EVs on hBMSCs behaviour. The influence on hydrogel-released EVs on hBMSCs (**A**) EV uptake (Scale bar = 20 µm), (**B**) proliferation, (**C**) migration, (**D**) H3K9 acetylation levels, (**F**) collagen production and (**E**,**F**) calcium deposition. Black staining indicates mineral nodules. Scale bar = 100 µm. Data are expressed as mean ± SD (*n* = 3). * *p* ≤ 0.05, ** *p* ≤ 0.01 and *** *p* ≤ 0.001.

To investigate the biological efficacy of hydrogel-released EVs, GelMA containing 1wt% LAP was utilised as this formulation exhibits a suitable balance in key properties such as shear-thinning behaviour, biocompatibility and EV release kinetics. The therapeutic efficacy of hydrogel-released EVs on hBMSCs behaviour was assessed using the transwell assay (Figure 1C). We observed the successful internalisation of hydrogel-released TSA-EVs by hBMSCs, with the labelled vesicles located primarily within the cytoplasm after 24 h of culture (Figure 5A). Hydrogel-released EVs significantly promoted hBMSCs proliferation in a time-dependent manner; cells incubated with the TSA-EV hydrogels eliciting a significantly enhanced DNA content when compared to the MO-EV treated (*p* ≤ 0.05–0.01) and the untreated cells (*p* ≤ 0.01) (Figure 5B). Similarly, the EV containing gels significantly increased hBMSCs migration when compared to the untreated cells following 3 days of culture (Figure 5C), with significantly enhanced migration observed in the TSA-EV groups (*p* ≤ 0.01–0.001).

Additionally, EV treated hBMSCs elicited enhanced H3K9 acetylation when compared to the untreated cells after 7 days of culture (Figure 5D). The TSA-EVs gels significantly promoted hBMSCs acetylation levels when compared to the MO-EVs (1.32-fold) (*p* ≤ 0.01) and the untreated cells (1.61-fold) (*p* ≤ 0.001). The osteoinductive potency of hydrogel-released TSA-EVs on hBMSCs was evaluated by assessing calcium deposition (Figure 5E,F). The TSA-EV treated cells elicited a substantial increase in alizarin red staining for calcium deposition when compared to the MO-EVs treated and untreated cells, with enhanced quantity of mineralised nodule formations observed (black staining). Quantitative analysis revealed that the TSA-EV treated hBMSCs exhibited a significant increase in extracellular matrix calcium deposition when compared to the MO-EV treated (1.87-fold) (*p* ≤ 0.001) and untreated cells (5.5-fold) after 21 days osteoinduction (*p* ≤ 0.001).

### 2.4. TSA-EVs Promote hBMSCs Extracellular Matrix Mineralisation within the GelMA-LAP Hydrogel

**Figure 6:**
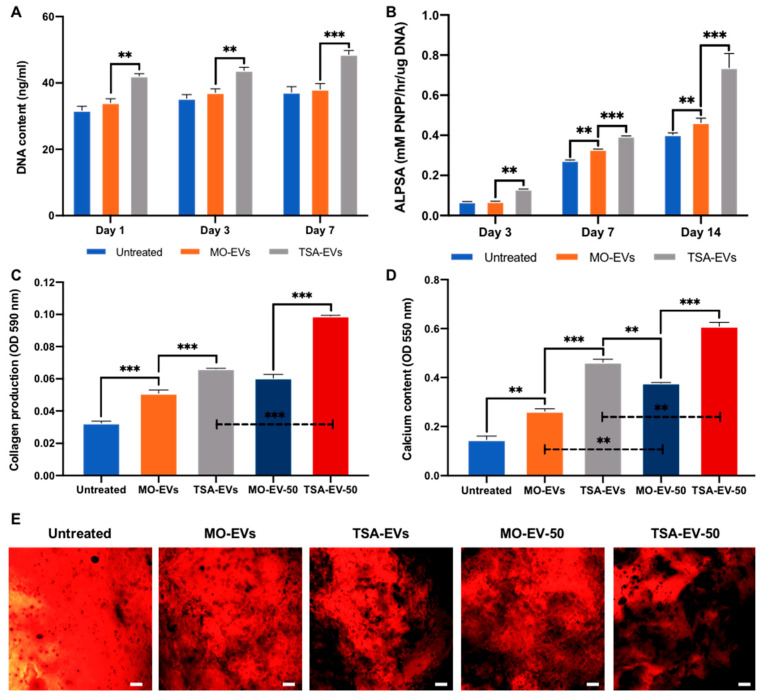
The effects of TSA-EVs on hBMSCs mineralisation within the GelMA-LAP hydrogel. (**A**) The influence of TSA-EVs on hBMSCs proliferation within the GelMA-LAP hydrogel during basal culture. The effect of TSA-EVs on hBMSCs (**B**) ALP activity, (**C**) collagen production and (**D**,**E**) calcium deposition following 21 days osteogenic culture. Black staining indicates mineral nodules. Scale bar = 100 µm. (MO-EV, TSA-EV; 10 µg/mL) (MO-EV-50, TSA-EV-50; 50 µg/mL). Data are expressed as mean ± SD (*n* = 3). ** *p* ≤ 0.01 and *** *p* ≤ 0.001.

The influence of TSA-EVs on encapsulated hBMSCs behaviour was initially evaluated by assessing proliferation. TSA-EV containing gels exhibited a significant increase in hBMSCs proliferation in a time-dependent manner when compared to the MO-EVs and EV-free groups (*p* ≤ 0.01–0.001) (Figure 6A). The effects of TSA-EVs on hBMSCs extracellular matrix mineralisation within the GelMA-LAP hydrogel was evaluated by assessing ALP activity, collagen production and calcium deposition. ALP activity was significantly enhanced in hBMSCs within the TSA-EVs gels when compared to the MO-EVs treated (1.89, 1.21, 1.61-fold) and the EV-free groups (1.94, 1.48, 1.85-fold) at day 3, 7 and 14 of osteogenic culture (*p* ≤ 0.01–0.001) (Figure 6B). Picrosirius red staining was conducted to evaluate EV-induced hBMSCs collagen production within the GelMA-LAP hydrogel (Figure 6C). The MO-EVs (1.57-fold) and TSA-EV treated (2.03-fold) groups elicited a significant increase in collagen content compared to the untreated control at day 21 (*p* ≤ 0.001). The TSA-EV treated group exhibited a 1.3-fold enhancement in collagen production when compared to the MO-EV treated cells (*p* ≤ 0.001). An EV dose-dependent increase in collagen content was observed, where 50 μg/mL EV treatment improved hBMSCs collagen production when compared to the 10 μg/mL EV treated groups (MO-EV-50 vs. MO-EVs, 1.19-fold (*p* > 0.05)) (TSA-EV-50 vs. TSA-EV, 1.5-fold (*p* ≤ 0.001)). The TSA-EV-50 group elicited a 1.65-fold increase in collagen production when compared to the MO-EV-50 group (*p* ≤ 0.001). TSA-EV containing gels exhibited a significant increase in alizarin red staining for calcium deposition when compared the MO-EVs treated (1.78-fold) and untreated control (3.14-fold) (*p* ≤ 0.001) (Figure 6D). Moreover, MO-EV-50 and TSA-EV-50 groups further improved hBMSCs calcium deposition when compared to the MO-EV and TSA-EV groups (MO-EV-50 vs. MO-EVs, 1.44-fold (*p* ≤ 0.01)) (TSA-EV vs. TSA-EV-50, 1.33-fold (*p* ≤ 0.01). The TSA-EV-50 gels displayed a 1.62-fold enhancement in calcium content when compared to the MO-EV-50 group (*p* ≤ 0.001). Interestingly, the TSA-EV group elicited a 1.23-fold significant increase in hBMSCs calcium deposition when compared to the MO-EV-50 group (*p* ≤ 0.01). Moreover, the TSA-EV-50 group displayed an increased quantity of mineralised nodule-like formations (black staining) (Figure 6E).

## 3. Discussion

A growing body of evidence has demonstrated the potential of harnessing EVs as novel acellular tools to promote bone regeneration [24,26,32]. However, issues associated with the therapeutic potency of vesicles has hindered their clinical potential. Several engineering strategies have been employed to enhance the translation of EV-based therapies to the clinical setting [15]. Previously we reported that altering the epigenome of mineralising osteoblasts substantially augmented the secreted EVs osteoinductive potency, thus providing a novel engineering strategy to enhanced EVs efficacy for bone repair [32]. The next logical step to facilitate the translation of epigenetically-modified EVs to the clinical setting is to evaluate their efficacy in situ. Although EV potency has been assessed in vivo [52,53], their short half-life hinders their therapeutic efficacy and ultimately tissue repair. Thus, there is a significant need to control the release kinetics of EVs in situ, to promote their ability to enhance bone formation. Therefore, in this present study, we investigated the development of an injectable nanocomposite GelMA hydrogel to facilitate TSA-EV induced bone regeneration.

GelMA hydrogels have been utilised for numerous tissue engineering applications due to its high biocompatibility, biodegradability and photosensitivity [40,41]. Although employed for different bone tissue engineering applications [54], the inherent lack of mechanical strength for GelMA hinders its application for load-bearing tissues. In order to improve GelMA utility for bone augmentation strategies, the addition of nanoclays have been described to augment the physical characteristics of the hydrogel. In this study, the influence of LAP on GelMA physicochemical properties were initially evaluated. We reported that LAP substantially enhanced the compressive modulus of the crosslinked hydrogel in a concentration-dependent manner, consistent with previously published studies [55,56]. As LAP exhibits both positive rim charge and negative surface charge, this allows for the strong electrostatic interactions within polymers to form physical crosslinked networks [57]. Moreover, it has been reported that nanosilicates are able to accelerate the gelation of polysaccharides through hydrogen bonding [58], providing the hydrogel with superior mechanical properties. The capability of nanosilicates to enhance GelMA physical properties, improves its clinical applicability and structural properties in situ, whilst also providing an osteoinductive microenvironment due to increased matrix stiffness [59]. In addition to exhibiting enhanced mechanical strength, it is highly favourable for EV-based biomaterials to elicit thixotropic behaviour. Biomaterials that display shear-thinning properties induce less stress to biological components within the hydrogel, such as cell or EVs, ultimately protecting their integrity and maximising their therapeutic efficacy [60]. Our findings showed that nanosilicate inclusion enhanced the shear-thinning properties of the hydrogel prior to crosslinking in a dose-dependent manner, consistent with findings in the literature [40,55]. We further demonstrated the LAP-induced shear-thinning behaviour promoted the shape fidelity of GelMA-LAP following 3D printing. These findings correlated with the influence of nanosilicates on improving the printability of numerous different polymers in the literature [60,61,62]. The enhanced shear-thinning behaviour and shape-fidelity provided by nanosilicates, significantly improves the efficacy of GelMA hydrogels for minimally invasive delivery via injection in addition to 3D printing, increasing its clinical utility. Taken together, these findings demonstrate the ability to control the shear-thinning, stiffness and shape-fidelity of GelMA hydrogels through the addition of LAP, ultimately enhancing the clinical efficacy of GelMA hydrogels for bone tissue engineering applications.

Several studies have reported the improved retention of EVs at the site of injury through delivery within biomaterial systems [52,53], however, there has been limited investigations on the role the delivery device plays on EV-induced tissue formation. GelMA hydrogels have been extensively utilised for bone tissue engineering applications due to their biocompatible nature and crosslinking capability [63,64]. Although well utilised, GelMA itself is not osteoinductive, thus there is growing research investigating the incorporation of additives to improve the biomaterials potential for bone repair. Previously, we reported the importance of delivering EVs with an osteoinductive vehicle to facilitate vesicle-induced mineralisation [65,66], thus emphasising the importance of biomaterial osteoinductivity of promoting EV functionality. The use of nanosilicates have been increasingly explored for bone tissue engineering applications due to the osteoinductive potential of its degradation products including lithium, magnesium and orthosilicic acid [67,68,69]. Thus, LAP could provide an osteoinductive environment to promote EV-induced mineralisation. Initially, we showed that LAP caused a dose-dependent effect on hBMSCs viability within the GelMA hydrogel. These results correlated with several studies in the literature indicating the influence of increased LAP concentration on the hydrogels porosity and mechanical properties, ultimately impacting proliferation and viability [70,71,72]. Importantly, we showed that nanosilicate inclusion significantly promoted hBMSCs mineralisation when compared to the LAP-free gel, consistent with reports in the literature [55]. This is likely due to the osteogenic potential of LAP degradation productions, in addition to the nanosilicate effects on construct stiffness, a key physical parameter influencing osteoinduction [59,73]. Moreover, there is growing evidence indicating the influence of cellular substrates on the biological efficacy of their secreted EVs, as vesicles are essentially fingerprints of their parental cell [74]. For example, 3D printed titanium scaffolds exhibiting a triangular pore shape accelerated osteoblast mineralisation when compared to square pore constructs. Osteoblast-derived EVs acquired from triangle pore scaffolds significantly increased hBMSCs osteogenic differentiation and mineralisation when compared to EVs acquired from other scaffold designs [26]. Therefore, in this study it is likely the cells within the LAP-containing gels release EVs with enhanced osteoinductive efficacy, promoting mineralisation in an autocrine/paracrine manner, however, this would require further investigation. Importantly, we showed that the introduction of osteoblast-derived EVs further improved hBMSCs mineralisation capacity within the GelMA-LAP hydrogel, suggesting the compatibility of combining pro-regenerative EVs within this biomaterial system. These results highlight the impact of LAP incorporation in improving the osteoinductivity of GelMA hydrogels, ultimately providing a viable platform to support EV-induced mineralisation.

In the repair of critical-sized bone fractures, the recruitment of endogenous cells into the defect site is vitally important for successful tissue regeneration [75]. Due to the diverse biological cargo that EVs possess, studies have demonstrated the role of these nano-sized vesicles in cellular recruitment [76,77]. Thus, there is an advantage for an EV-functionalised biomaterial to release a proportion of the delivered EVs to stimulate endogenous cell recruitment into the defect. This is particularly important if delivered as an acellular treatment. GelMA hydrogels have been reported to facilitate EV delivery in several applications [42,78]. For example, Tang et al. demonstrated EV-laden GelMA hydrogels improved the cardiac function of mice following myocardial infarction when compared to EVs delivered in saline solution [78]. Although encouraging results have been shown, the vesicle release kinetics from these systems likely relying on polymer concentration, which also impacts tissue regeneration. Due to the issues with EV retention in vivo [33], there is growing precedence to develop biomaterials that can facilitate the delivery and release profile of these pro-osteogenic bioactive factors in situ. Nanosilicates have demonstrated their ability to control growth factor delivery through favourable protein interactions, their discotic charged surface and high-surface to volume-ratio [46,79,80], which could be exploited to control EV release kinetics in vivo. Our findings showed a LAP dose-dependent effect on EV release from the GelMA hydrogels, where at day 7, GelMA alone exhibited a 4.1- and 25-fold significant increase in the quantity of vesicles released when compared to the 1wt% and 2wt% LAP groups, respectively. Hu et al. similarly reported that enhanced retention of MSC-EVs when combined with a GelMA/nanoclay hydrogel for cartilage regeneration [81]. The improved retention of EVs with LAP is likely due to the nanosilicates clay–protein electrostatic interactions facilitating increased immobilisation of these vesicles within the hydrogel. Moreover, it has been shown that nanosilicate inclusion reduced the porosity of GelMA hydrogels [56], possibly contributing to enhanced EV retention. In addition to assessing EV release kinetics from the GelMA-LAP gel, it is critical to determine whether the hydrogel-released EVs retain their biological potency. For this analysis, GelMA containing 1wt% LAP was utilised due to its shear-thinning and biocompatible behaviour, whilst exhibiting controlled EV release kinetics when compared to the other formulations tested, an important aspect to stimulate endogenous cell recruitment. Our findings showed that EVs released from the GelMA-LAP hydrogel, promoted the migration and proliferation of hBMSCs, essential characteristics for endogenous cell recruitment. TSA-EVs significantly promoted proliferation and migration when compared to the MO-EV and the EV-free gel, consistent with their efficacy observed in 2D in vitro culture [32]. Moreover, hBMSCs treated with hydrogel-released TSA-EVs elicited a significant increase in histone acetylation levels. Hyperacetylation has been reported to enhance the differentiation capacity of cells due to chromatin remodelling and transcription factor activation [82,83]. Previously proteomic analysis highlighted the significant enrichment of proteins involved in transcriptional regulation and epigenetic modification within TSA-EVs [32]. Therefore, the increased histone acetylation levels induced by TSA-EVs treatment, likely imbued hBMSCs with enhanced differentiation capacity due to the delivery of epigenetic modifying proteins. Importantly, our findings showed that TSA-EVs were able to substantially enhance hBMSCs extracellular matrix mineralisation when released from the GelMA nanocomposite hydrogel, consistent with the observations in 2D in vitro culture [32]. Additionally, having confirmed a similar quantity of MO-EVs and TSA-EVs released from these GelMA-LAP hydrogels, the enhanced stimulation observed at recipient hBMSCs is likely due to TSA-EVs increased biological potency rather than differences in the concentration of nanoparticles released. Taken together, these findings demonstrate the GelMA-LAP system controlled the EV release kinetics and preserved the biological potency of these epigenetically modified EVs when released from the hydrogel, indicating the GelMA-LAP system provides a suitable vehicle to deliver TSA-EVs without sacrificing their functionality.

There is a growing body of evidence demonstrating the delivery of EV-functionalised biomaterials to promote bone regeneration [84,85], however, the vehicle employed often does not support EV-induced tissue regeneration. For example, Holkar et al. reported the potential of an alginate hydrogel loaded with osteoblasts and their EVs for bone tissue engineering applications [85], however, alginate itself is osteogenically inert. The importance of the delivery vehicle on EV functionality was demonstrated by Davies et al., where osteoblast-derived EVs exhibited substantially enhanced mineralisation potency when delivered in mineralising medium compared to non-mineralising medium [65]. Therefore, delivering pro-osteogenic EVs with a biomaterial system that facilitates EV-induced tissue regeneration is vital to maximise the therapeutic response of these nanoparticles in vivo. In this study, having developed a nanocomposite formulation that exhibits sufficient physiochemical and osteoinductive properties, whilst eliciting controlled EV release kinetics, the next logical step was to evaluate TSA-EV efficacy in stimulating hydrogel-encapsulated hBMSCs mineralisation. Our findings showed that EV-loaded GelMA nanocomposites promoted the proliferation of encapsulated hBMSCs, with the TSA-EVs further improving proliferation. The significantly enhanced proliferation within the TSA-EV group and not the MO-EVs hydrogel, indicates the importance of the epigenetic reprogramming strategy in maximising the potency of these vesicles within this system. Importantly, our results showed that TSA-EV functionalised gels significantly enhanced hBMSCs osteogenic differentiation and extracellular mineralisation through increased ALP activity (1.61-fold), collagen production (1.3-fold) and calcium deposition (1.78-fold) when compared to the MO-EV group. Moreover, TSA-EVs elicited an enhanced dose-dependent increase in hBMSCs extracellular matrix collagen production and calcium depositions when compared to the MO-EV hydrogels, thus providing greater evidence regarding TSA-EVs enhanced osteoinductive potency. The effects of TSA-EVs on hBMSCs proliferation and mineralisation within the hydrogel, were consistent with the EV hydrogel release results and previously published reports [32]. These findings indicate the encapsulation of TSA-EVs within the GelMA-LAP hydrogel did not adversely impact the osteoinductive potency of these epigenetically enhanced EVs within the hydrogel system. Interestingly, we observed a greater degree of enhancement in TSA-EVs induced hBMSCs mineralisation within the GelMA-LAP hydrogel when compared to the TSA-EV treatment in 2D culture [32]. Within the nanocomposite hydrogel, TSA-EVs elicited a 2.03 and 3.14-fold increase in hBMSCs collagen production and calcium deposition when compared to the untreated cells, whilst TSA-EVs induced a 1.68 and 1.52-fold enhancement in collagen and calcium production in 2D culture. A similar trend was observed when hBMSCs, treated with the HDACi MI192, elicited a 1.43-fold enhanced mineralisation when cultured within the 3D bio-assembled microtissue construct in comparison to 2D culture [29]. These findings suggest the GelMA-LAP 3D microenvironment augments TSA-EVs efficacy in stimulating mineralisation when compared to 2D monolayer culture.

The increased TSA-EV osteoinductive efficacy observed within the GelMA-LAP hydrogel is likely due to a combination of several factors. It is known that cells suspended within a 3D matrix elicit an altered cellular response to physical and chemical stimulation [86,87]. Thus, it is probable hBMSCs encapsulated within the GelMA-LAP hydrogel were more receptive to the osteoinductive stimulation induced by TSA-EVs within the construct when compared to 2D cultured hBMSCs. Additionally, we previously reported that the culture platform influences the transcriptional permissiveness of cells. Triangular pore titanium scaffolds substantially enhanced osteoblast histone acetylation, resulting in increased mineralisation [26]. Thus, GelMA-LAP 3D microenvironment likely altered the epigenetic landscape of encapsulated hBMSCs, priming them with enhanced differentiation capacity when compared to 2D cultured cells. Finally, due to the 3D microenvironment provided by the GelMA-LAP hydrogel and the hBMSCs produced ECM, this likely influenced the sequestering of bioactive factors within the secretome [4], such as EVs, further facilitating mineralisation within the GelMA-LAP hydrogel. Taken together, these findings demonstrate the GelMA-LAP hydrogel provides a viable platform to facilitate the delivery and osteoinductive potency of epigenetically enhanced EVs, as a novel acellular strategy to stimulate bone regeneration. As the physical and biological performance of biomaterials are intrinsically linked and due to the disparity between the in vitro and in vivo setting, the next logical step to evaluate the therapeutic potency of this EV-functionalised hydrogel, is to investigate its capacity to promote bone fracture healing within a more physiologically relevant environment in future in vivo studies. Bone regeneration is a multi-faceted process synergistically involving angiogenesis, osteogenesis and innervation. Therefore, employing more physiological relevant models in vivo (i.e., subcutaneous implantation and femoral fracture repair) would provide increased pre-clinical evidence regarding the therapeutic efficacy of this EV-functionalised hydrogel targeted for bone repair.

## 4. Materials and Methods

### 4.1. GelMA and Nanocomposite Synthesis

Type A porcine skin gelatin (Sigma-Aldrich, Gillingham, UK) was mixed at 10% (wt%) into Dulbecco’s phosphate buffered saline (PBS, Lonza, Manchester, UK) until fully dissolved. Methacrylic anhydride (Sigma-Aldrich, Gillingham, UK) was added (0.6 g/mL) to gelatin solution under stirred conditions at 50 °C and incubated for 1 h. The mixture was dialyzed against distilled water using 12–14 kDa cut-off dialysis tubing (Thermo Scientific, Paisley, UK) for 2–3 days at 40 °C to remove salts and methacrylic acid. After dialysis, the GelMA solution was diluted to 2% (*w*/*v*) and the pH adjusted to 7.4 using 1 mM sodium hydroxide solution (Sigma-Aldrich, Gillingham, UK). Then, the solution was sterile filtered and lyophilised for 2 days. Lyophilised GelMA was stored at –80 °C until further use.

The nanocomposite hydrogel was fabricated by dispersing sterile Laponite-XLG XR (BYK Additives & Instruments, Widnes, UK) in sterile deionised water (0, 0.5, 1, and 2 wt%) with stirring at 300 rpm for 3 h. Sterile GelMA (5wt%) was allowed to dissolve in the LAP suspension overnight at 37 °C.

### 4.2. Fabrication of GelMA-LAP Hydrogel

The photoinitiators (1 mM Tris (2,2′-bipyridyl) dichlororuthenium (II) hexahydrate (Ru) (Sigma-Aldrich, Gillingham, UK) and 10 mM Sodium persulfate (SPS) (Sigma-Aldrich, Gillingham, UK)) were added to different GelMA-LAP formulations and 60 μL of the solution was transferred into silicone moulds (Ø 5 mm × 2 mm), covered with a glass slide. Hydrogels were then crosslinked for 5 min using visible light (Knightsbridge FLF Floodlight, RS, Corby, UK).

### 4.3. Rheology

The viscoelastic behaviour of the nanocomposite hydrogel pre-polymer solutions was assessed using the Kinexus Pro+ rheometer (Malvern Instruments, Malvern, UK). An oscillatory strain sweep was performed between 1% and 100% strain at 0.1 Hz frequency and 37 °C. Toothpaste was used as the positive control, since it exhibits shear-thinning behaviour.

### 4.4. Mechanical Testing

The Young’s modulus of the crosslinked nanocomposite gels was assessed via cyclic testing using a Instron 5542 mechanical tester (Instron, Norwood, MA, USA) with a 100 N load cell. Cylindrical hydrogels (Ø8 mm × 2 mm) were prepared as previously described and incubated in Phosphate buffered saline (PBS, Lonza, Manchester, UK) for 4 h prior to testing. Compression testing was performed at a rate of 1 mm/min to a maximum strain of 60% by performing 8 cycles of loading/unloading. The load (N) and compressive strain (mm) was assessed using the Bluehill 3 software. The Young’s modulus was calculated from the slope of the linear region of the stress (kPa)/strain (mm/mm) curves for the 8th cycle. Samples were tested in triplicate for each condition.

### 4.5. Fabrication of 3D Printed GelMA-LAP Construct

To evaluate bioinks shape fidelity, the extrusion-based 3D printing of GelMA or GelMA-LAP (1wt%) bioinks was performed using a 3D Discovery bioprinter (RegenHU, Villaz-Saint-Pierre, Switzerland). Pre-polymer solutions were fabricated at elevated temperatures and loaded into a 3D Discovery bioprinter and protected from light. Computer aided design models were created using the BIOCAD software (3DDiscoverGS, RegenHU, Villaz-Saint-Pierre, Switzerland). The bioinks were extruded using a computer-aided syringe dispenser with a 20-gauge needle, a feed rate of 3 mm/s and pressure of 10 bar. Constructs were printed within a 6 well plate and irradiated for 5 min with visible light using the same Ru/SPS photoinitiator concentrations as described above.

### 4.6. Cell Culture and Reagents

MC3T3 murine pre-osteoblasts were purchased from American Type Culture Collection (ATCC, Teddington, UK) and hBMSCs were acquired from Lonza (Lonza, Manchester, UK). Basal culture media consisted of minimal essential medium (α-MEM; Sigma-Aldrich, Gillingham, UK) supplemented with 10% foetal bovine serum (FBS), 1% penicillin/streptomycin (Sigma-Aldrich, Gillingham, UK) and L-glutamine (Sigma-Aldrich, Gillingham, UK). hBMSCs were used at passage 4. Mineralisation medium comprised of basal culture media supplemented with 10 mM β-glycerophosphate (Sigma-Aldrich, Gillingham, UK) and 50 μg/mL L-ascorbic acid (Sigma-Aldrich, Gillingham, UK). Culture medium utilised for EV isolation and dosing was depleted of FBS-derived EVs by ultracentrifugation at 120,000× *g* for 16 h prior to use.

### 4.7. The Biological Efficacy of GelMA Nanocomposite

The influence of LAP concentration on GelMA biological efficacy was initially investigated by evaluating proliferation. Briefly, hBMSCs were mixed at low density (5 × 10^5^ cell/mL) in the hydrogel prior to gelation. Following sol-gel transition, hydrogels were cultured in basal medium for 2 weeks with media changes every 3 days. At each time point, Alamarblue reagent (Thermo Scientific, Paisley, UK) was added and incubated for 4 h at 37 °C. Following which, fluorescence readings were acquired using a SPARK spectrophotometer (TECAN, Männedorf, Switzerland) at an excitation/emission wavelength of 540/590 nm, respectively. Percentage cell viabilities were calculated taking only the GelMA group (0wt%) as 100%.

To evaluate the hydrogel effect on osteoinduction, hBMSCs were mixed at high density (1 × 10^6^ cell/mL) in the hydrogel prior to gelation. Following sol-gel transition, hydrogels were incubated in basal medium for 24 h. After this time, the media was replaced with osteogenic medium and gels were cultured for 2 weeks, with media changes occurring every 3 days.

### 4.8. EV Isolation and Characterisation

#### 4.8.1. EV Isolation

The manufacture of TSA-EVs was conducted following previously published protocols [32]. Briefly, osteoblasts were cultured at scale in T175 culture flasks (Sarstedt, Leicester, UK) and medium isolated every two days. Cells were cultured in osteogenic medium supplemented with/without 5 nM TSA for 14 days. EVs were isolated from conditioned medium (400 mL) by differential centrifugation: 2000× *g* for 20 min, 10,000× *g* for 30 min and 120,000× *g* for 70 min to pellet EVs [32]. The supernatant was removed, and the pellet was washed in sterile PBS and centrifuged at 120,000× *g* for 70 min and the resultant pellet was re-suspended in 200 μL PBS. All ultracentrifugation steps were performed utilising the Sorvall WX Ultra Series Ultracentrifuge (Thermo Scientific, Paisley, UK) and a Fiberlite, F50L-8×39 fixed angle rotor (Piramoon Technologies Inc., Santa Clara, CA, USA). EV characterisation was conducted following guidelines published in the Minimal Information for Studies of Extracellular Vesicles 2018 [88].

#### 4.8.2. Transmission Electron Microscopy (TEM)

EV imaging was conducted via a JEOL JEM1400 transmission electron microscope (TEM) coupled with an AMT XR80 digital acquisition system. Samples were physisorbed to 200 mesh carbon-coated copper formvar grids (Agar Scientific, Stansted, UK) and negatively stained with 1% uranyl acetate.

#### 4.8.3. EV Particle Size, Concentration and Tetraspanin Analysis

A NanoAnalyzer U30 instrument (NanoFCM Inc., Nottingham, UK) equipped with dual 488/640 nm lasers and single-photon counting avalanche photodiode detections (SPCM APDs) was used for simultaneous detection of side scatter (SSC) and fluorescence of individual particles. The concentration of samples was determined by comparison to 250 nm silica nanoparticles of known concentration to calibrate the sample flow rate. EV isolates were sized according to standard operating procedures using a proprietary 4-modal silica nanosphere cocktail (NanoFCM Inc., S16M-Exo). Using the NanoFCM software (NanoFCM Profession V1.8), a standard curve was generated based on the side scattering intensity of the four different silica particle populations. Measurements were taken over a 1 min interval at a sampling pressure of 1.0 kPa, maintained by an air-based pressure module. All samples were diluted to attain a particle count within the optimal range of 2000–12,000/min. Particle concentration and size were calculated using the NanoFCM software (NanoFCM Profession V1.8, NanoFCM, Nottingham, UK).

For EV tetraspanin phenotyping, the following antibodies were used: APC-conjugated anti-mouse CD63 (clone NVG-2; Biolegend, San Diego, CA, USA), APC-conjugated anti-mouse CD9 (clone EM-04; Abcam, Cambridge, UK) and APC-conjugated anti-mouse CD81 (clone EAT-2; Biolegend, San Diego, CA, USA). EV sample was diluted to 1 × 10^10^ particles/mL in PBS and 9 µL was mixed with 1 µL of conjugated antibody (single or mixed cocktail), before incubation for 30 min at room temperature. Incubation concentration ratio for single antibodies was 1:50 (1 µL of 1:5 in PBS) and 1:150 for the cocktail of 3 antibodies (1 µL of 1:5 of mixed antibody cocktail). After incubation, the mixture was diluted in PBS to 1 × 10^8^–1 × 10^9^ particles/mL for immediate phenotypic analysis.

Dynamic Light Scattering (DLS) (Zetasizer Nano ZS, Malvern Instruments, Malvern, UK) was used to analyse zeta potential. Total EV protein concentration was determined using the Pierce BCA protein assay kit (Thermo Scientific, Paisley, UK).

### 4.9. EV Release Kinetics from Hydrogels

The in vitro release kinetics of EVs within the GelMA-LAP hydrogel (EVs at 100 µg/mL) was assessed as previously reported [36]. Briefly, EV-functionalised gels were incubated in sterile PBS at 37 °C. At days 1, 3, 5 and 7, the receiving medium was collected, and replaced by an equal volume of fresh PBS. The EV concentration in the collected medium was evaluated using the CD63 ExoELISA-ULTRA complete kit (System Biosciences, Palo Alto, CA, USA) following the manufacturer’s protocol.

### 4.10. The Impact of Hydrogel-Released EVs on hBMSCs Proliferation, Migration and Mineralisation

#### 4.10.1. EV Cell Uptake

EVs were labelled using Cell Mask^TM^ Deep Red Plasma Membrane Stain, 1:1000 in PBS, (Thermo Scientific, Paisley, UK) and incubated for 10 min. Labelled EVs were washed twice with PBS via ultracentrifugation at 120,000× *g* for 70 min, then loaded within the GelMA-LAP hydrogel before gelation. hBMSCs were seeded at 3 × 10^3^ cells/cm^2^ in a 48 well plate for 24 h, then media was replaced with fresh basal medium and transwell inserts (0.4 µm pore size, Greiner Bio-One, Stonehouse, UK) containing Cell Mask^TM^-labelled EVs encapsulated within the hydrogel. Cells cultured with EV-free hydrogels were used as the control.

#### 4.10.2. Proliferation Assay

hBMSCs were plated at low density (1 × 10^4^ cells/cm^2^) in basal medium within a 48 well plate. After 24 h, media was replaced with fresh basal medium and transwell inserts (0.4 µm pore size, Greiner Bio-One, Stonehouse, UK) containing EV-functionalised hydrogels were placed into each well. Media was replaced every 3 days. DNA content was assessed using the PicoGreen (Life Technologies, Paisley, UK) according to the manufacturer’s protocol. Cells cultured with EV-free hydrogels were used as the control.

#### 4.10.3. Migration Assay

The migration rates were calculated by performing the wound healing assay. Briefly, cells at a density of 3 × 10^4^ cells/cm^2^ in a 48 well plate were plated and allowed to adhere for 24 h. A scratch was applied with a 200 µL pipette tip and the width was measured as the baseline. Cells were incubated with transwell inserts (0.4 µm pore size, Greiner Bio-One, Stonehouse, UK) containing EV-functionalised hydrogels for 3 days. Cells cultured with EV-free hydrogels were used as the control. The rate of wound closure from day 0 was assessed using light microscopy (EVOS XL Core, Invitrogen, Paisley, UK).

#### 4.10.4. H3K9 Acetylation Assay

Cells were cultured in a 48 well plate (3 × 10^4^ cells/cm^2^) in basal medium. After 24 h, media was replaced with fresh basal medium and transwell inserts (0.4 µm pore size, Greiner Bio-One, Stonehouse, UK) containing EV-functionalised hydrogels, were placed into each well. Following 7 days of culture, the detection of H3K9 acetylation was performed using the EpiQuik^TM^ In Situ Histone H3-K9 Acetylation Assay Kit (Epigentek, Farmingdale, NY, USA) according to the manufacturer’s protocol. The absorbance was read in a SPARK spectrophotometer (TECAN, Männedorf Swizerland) at 450 nm. Histone acetylation was normalised with DNA content. Cells cultured with EV-free hydrogels were used as the control.

#### 4.10.5. Osteoinduction

hBMSCs were seeded in a 48 well plates at a density of 3 × 10^4^ cells/cm^2^ in basal medium and incubated for 24 h. The media was replaced with mineralisation medium and transwell inserts, (0.4 µm pore size, Greiner Bio-One, Stonehouse, UK) containing EV-functionalised hydrogels, were placed into each well for 21 days Mineralisation medium changes were performed every 48 h. Cells incubated with EV-free hydrogels were used as the untreated control.

### 4.11. TSA-EV Functionalised Hydrogels on hBMSCs Proliferation and Mineralisation

The proliferation of hBMSCs within the EV-functionalised nanocomposite hydrogel was assessed. Briefly, hBMSCs (5 × 10^5^ cell/mL) were mixed with the EV-functionalised hydrogel prior to photo-crosslinking. The proliferation of cell-laden EV-hydrogels was assessed via quantifying DNA content following culture in basal medium for 7 days. DNA content was assessed using PicoGreen (Life Technologies, Paisley, UK) according to the manufacturer’s protocol.

The capacity of EV-functionalised hydrogels to stimulate encapsulated hBMSCs (1 × 10^6^ cell/mL) osteogenic differentiation and mineralisation was evaluated after culture in osteogenic medium for 21 days. Osteogenic differentiation was assessed by quantifying alkaline phosphatase activity, collagen production and mineral deposition, detailed below.

### 4.12. Alkaline Phosphatase (ALP) Activity

ALP activity was determined using the 4-nitrophenyl colourimetric phosphate liquid assay (pNPP, Sigma-Aldrich, Gillingham, UK) as previously described [29]. Briefly, 10 μL of cell lysate was added to 90 μL of pNPP and incubated for 60 min at 37 °C. The absorbance at 405 nm was read on a SPARK spectrophotometer (TECAN, Männedorf, Switzerland). ALP activity was normalised with DNA content.

### 4.13. Collagen Production

Extracellular matrix collagen deposition was evaluated with picrosirius red staining. Briefly, samples were washed twice in PBS and fixed in 10% neutral buffered formalin (NBF, Cellpath, Newtown, UK) for 30 min, prior to staining with 0.1% sirius red in saturated picric acid (Sigma-Aldrich, Gillingham, UK) for 1 h. The unbound dye was removed by washing in 0.5 M acetic acid (Sigma-Aldrich, Gillingham, UK) followed by distilled water wash and left to air dry. To quantify collagen staining, 0.5 M sodium hydroxide (Sigma-Aldrich, Gillingham, UK) was used to elute the bound dye and absorbance were read at 590 nm using the SPARK spectrophotometer (TECAN, Männedorf, Switzerland).

### 4.14. Mineral Deposition

To evaluate mineralisation, calcium deposition was assessed via alizarin red staining. Samples were washed twice in PBS and fixed in 10% NBF (Cellpath, Newtown, UK) for 30 min. Following fixation, constructs were washed in distilled water and then incubated with alizarin red solution (Sigma-Aldrich, Gillingham, UK) for 10 min. The unbound dye was removed by washing in distilled water. Staining was visualised using light microscopy (EVOS XL Core, Invitrogen, Paisley, UK). For alizarin red quantification, samples were de-stained with 10% cetylpyridinium chloride (Sigma-Aldrich, Gillingham, UK) for 1 h and then absorbance were read at 550 nm using the SPARK spectrophotometer (TECAN, Männedorf, Switzerland).

### 4.15. Statistical Analysis

For all data presented, experiments were performed in triplicate. All statistical analysis was undertaken using ANOVA multiple comparisons test with Tukey modification using IBM SPSS software (IBM Analytics, version 21). *p* values equal to or lower than 0.05 was considered as significant. * *p* ≤ 0.05, ** *p* ≤ 0.01 *** *p* ≤ 0.001.

## 5. Conclusions

Taken together, we have demonstrated the development of a nanocomposite photocurable hydrogel functionalised with epigenetically activated pro-osteogenic EVs as a novel acellular tool to stimulate bone regeneration.

## Data Availability

The data presented in this study are available on request from the corresponding author.

## Supplementary Materials

The following supporting information can be downloaded at: https://www.mdpi.com/article/10.3390/ijms23020832/s1.
