# Supplementary material for: Controlled Release of Epigenetically-Enhanced Extracellular Vesicles from a GelMA/Nanoclay Composite Hydrogel to Promote Bone Repair
Source: Int J Mol Sci. 2022 Jan 13;23(2):832. doi: 10.3390/ijms23020832 (PMC8775793; doi:10.3390/ijms23020832)
Supplement: Supplementary file 1 [file ijms-23-00832-s001.zip › ijms-1531650-supplementary.pdf]

Supplementary Materials

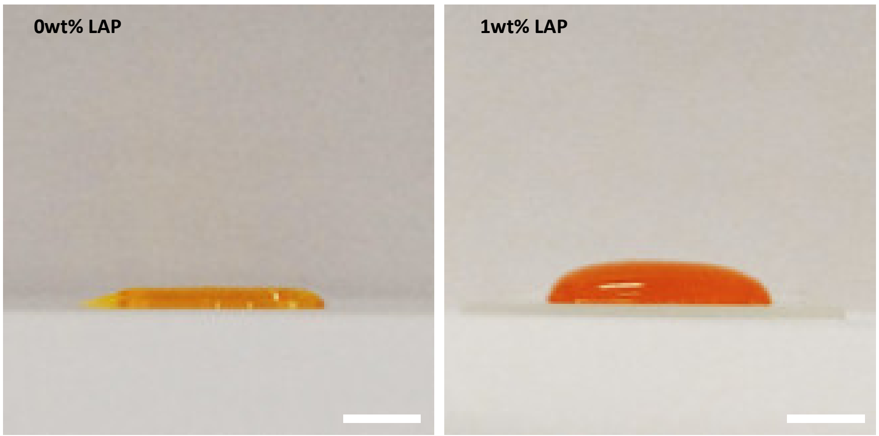

Supplementary Figure S1. 3D printed GelMA-LAP hydrogel integrity in the z-axis. GelMA with/without LAP were extruded as filaments (4 layers) to evaluated shape fidelity in the z-axis. Scale bar = 500 μm.

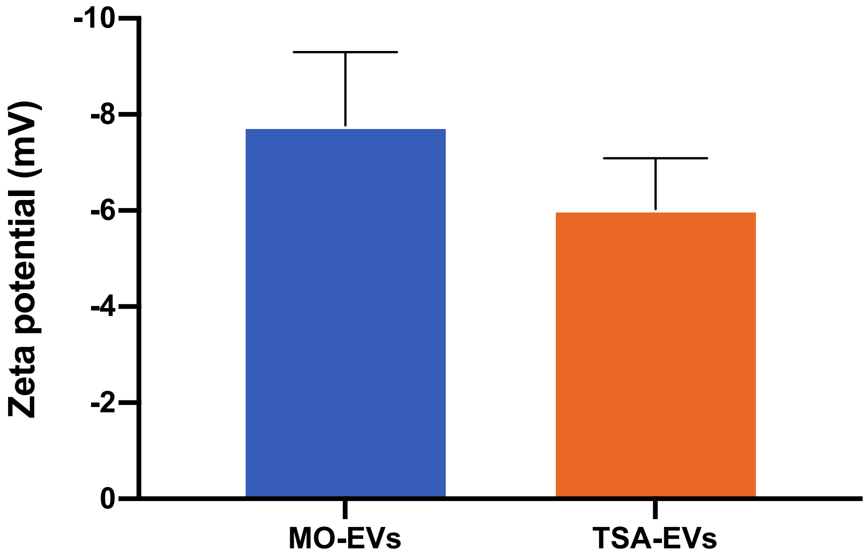

Supplementary Figure S2. Zeta potential of osteoblast-derived EVs. Data are expressed as mean ± SD (n = 3).

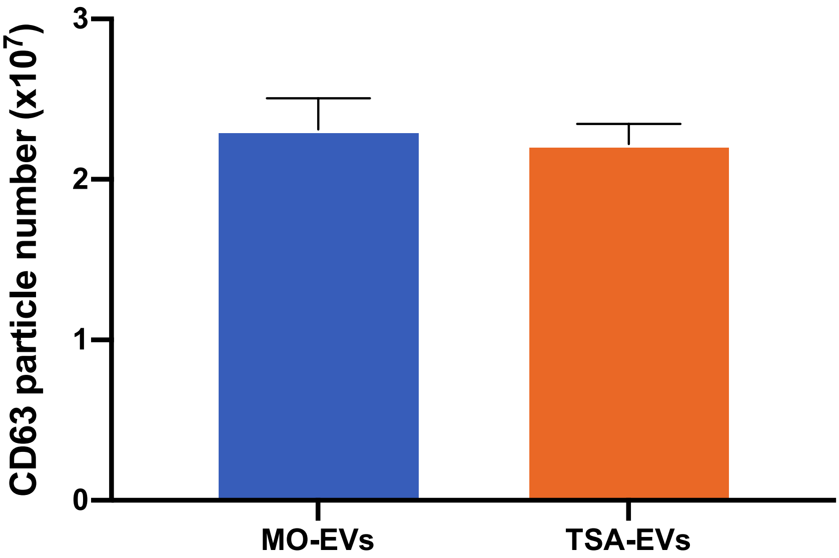

Supplementary Figure S3. Quantification of CD63 positive particles from TSA-EV or MO-EVs loaded GelMA hydrogel containing 1wt% LAP after 7 days incubation. Data are expressed as mean ± SD (n = 3).
